# The effect of educational intervention based on theory of planned behavior on behavioral responses of premenopausal women in prevention of osteoporosis

**DOI:** 10.1186/s12905-022-01953-1

**Published:** 2022-09-06

**Authors:** Ali Khani Jeihooni, Tayebeh Rakhshani, Zahra Khiyali, Mohammad Mehdi Ebrahimi, Pooyan Afzali Harsini

**Affiliations:** 1 Nutrition Research Center, Department of Public Health, School of Health, Shiraz University of Medical Sciences, Shiraz, Iran; 2 Departement of Public Health, School of Health, Fasa University of Medical Sciences, Fasa, Iran; 3 Department of Public Health, School of Health, Fasa University of Medical Sciences, Fasa, Iran; 4 Department of Public Health, School of Health, Kermanshah University of Medical Sciences, Kermanshah, Iran

**Keywords:** Osteoporosis, Education, Behavior, Women

## Abstract

**Background:**

Osteoporosis is one of the most prevalent bone diseases which is preventable. Implementing educational programs is an important step in prevention of chronic diseases in the community setting. One of the theories used for predicting behavior and performing educational intervention is theory of planned behavior (TPB) which predicts the intention of an individual toward doing a specific behavior. This study was conducted to assess the effect of educational intervention based on TPB on behavioral responses of premenopausal women in prevention from osteoporosis in Fasa city, Iran.

**Methods:**

This study is a quasi-experimental study performed on 200 women aging from 35 and 55 years who referred to health centers in Fasa city; iran in 2019. Simple random sampling was applied to assign participants to control and intervention groups (100 participant for each group). Data were gathered by a "valid" and "reliable" questionnaire arranged based on the constructs of TPB, nutrition performance and physical activity. An educational program on osteoporosis prevention was conducted. educational intervention was performed in six sessions through group discussions and educational films and booklet for experimental group and then the changes in the scores of the two groups were evaluated and compared. Obtained data were analyzed by SPSS-22 software through Chi-square, independent t-test, paired t-test and ANOVA tests.

**Results:**

The mean ages of studied participants in experimental and control groups were respectively 43.39 $\pm$ 5.20 and 42.94 $\pm$ 5.52. In experimental group, the average scores of knowledge [2 weeks (31.12 $\pm$ 4.20) and 2 months (39.04 $\pm$ 4.10) after educational intervention (p < 0.001)], constructs of theory of planned behavior [attitude construct: 2 weeks (89.32 $\pm$ 9.22) and 2 months (98.57 $\pm$ 9.13) after educational intervention (p < 0.001), Subjective norms construct: 2 weeks(88.39 $\pm$ 8.84) and 2 months (122.57 $\pm$ 8.58) after educational intervention (p < 0.001), Perceived behavioral control construct: 2 weeks (88.56 $\pm$ 8.38) and 2 months (120.15 $\pm$ 8.33) after educational intervention (p < 0.001), Behavioral intention construct: 2 weeks (54.44 ± 4.72) and 2 months (60.26 $\pm$ 4.12) after educational intervention (p < 0.001)], nutrition performance [2 weeks (19.88 ± 2.56) and 2 months (24.14 $\pm$ 2.36) after educational intervention (p < 0.001)] and physical activity [2 weeks (16.75 $\pm$ 1.42) and 2 months (18.94 $\pm$ 1.68) after educational intervention (p < 0.001)] had more significant enhancement than control group 2 weeks and 2 months after educational intervention.

**Discussion:**

TPB was effected in nutrition performance and physical activity in osteoporosis prevention of subjects. This theory can be used as a framework for designing and performing educational intervention for preventing osteoporosis and promoting women’s health.

## Background

Menopause is one of the stages of a woman's life that comes with both troubles and benefits. Because menopause is unavoidable and affects every woman, identifying its risk factors, diseases, and side effects is critical, which can be accomplished through health education [1]. Osteoporosis is one of the most severe consequences of our time [2], as it is a worldwide illness that causes bone density loss, changes in bone structure, and, as a result, the risk of bone fracture [3]. Fractures caused by inadequate bone density can result in chronic pain, disability, loss of independence, a decline in life quality, and an increase in the death rate [4]. DXA measurements of the hip and spine are used to establish or confirm an osteoporosis diagnosis, estimate future fracture risk, and monitor patients. Areal BMD is expressed in absolute terms of grams of mineral per square centimeter scanned (g/cm^2^) and as a connection to two norms: compared to the BMD of an age-, sex-, and ethnicity-matched reference population (Z-score) or compared to a young-adult reference population of the same sex (T-score). T-scores and Z-scores are calculated using the difference between the patient's BMD and the mean BMD of the reference population, divided by the standard deviation (SD) of the reference population. Peak bone mass is reached in early adulthood, followed by a reduction in BMD. The rate of bone loss rises in women during menopause and slows in older postmenopausal women and men. As seen in, an individual's BMD is expressed as the standard deviation above or below the mean BMD of the reference population. The WHO diagnostic categorization is used to determine BMD diagnoses of normal, low bone mass (osteopenia), osteoporosis, and severe or established osteoporosis. The WHO definition of osteoporosis based on BMD [Within 1 SD of the mean level for a young-adult reference population BMD (T-scores of 1.0 and above are included in Normal Classification, between 1.0 and 2.5 SD below the mean level for a young-adult reference population BMD Low bone mass (osteopenia) is defined as BMD (T-score between 1.0 and 2.5), which is 2.5 SD or more below the mean level for a young-adult reference population. BMD (T-score at or below 2.5) is included in the Osteoporosis categorization and 2.5 SD or more below the mean level for a young-adult reference population with fractures. Severe or established osteoporosis is defined as a BMD (T-score of 2.5 or less with one or more fractures) [5].

Women are more susceptible to osteoporosis than men. This condition affects more than half of women over the age of 50 [6]. Around the world, almost 200 million women suffer from osteoporosis [7, 8]. In general, the prevalence of osteopenia in premenopausal women ranges between 15 and 30 percent, while the prevalence of osteoporosis ranges between 0.1 and 3.2 percent [9]. In Iran, the prevalence of low bone density in women is 51%, and the prevalence of osteoporosis is 32%, with 32% in the spine's bones, 21% in the spinal cord, and 25% and 21% in the neck and hip joint, respectively [10]. Most variables, including age, gender, menopause, a family history of fracture, insufficient calcium in the diet, a lack of vitamin D, BMI, low physical activity, and thyroid function, are linked to changes in bone density [11–13], the quantity of calcium obtained through diet and walking are two essential factors in the prevention and treatment of osteoporosis [14]. If appropriate preventive measures are not implemented, the global cost of osteoporosis is expected to exceed $200 billion by 2040. Despite the rising frequency of osteoporosis in Iran, there is a lack of data on its true prevalence and accompanying burden [15].

investigated participants did not consider the observation of factors and prevention behaviors from osteoporosis [16, 17]. Therefore, performing educational programs for increasing knowledge and improving prevention behaviors are demanded. Educational programs include three main issues: Knowledge on osteoporosis, medicine and diet and exercising [18]. Because preventing inappropriate behavioral factors needs changing individual’s behavior [19], hence, health education and promotion patterns and theories can be efficient on osteoporosis prevention [20].

Choosing a suitable model or theory is one of the most crucial activities in educational interventions. Health education is meaningless without good programming [21]. The application of theories for osteoporosis prevention education can assist in identifying areas that require greater emphasis within programs. TPB is a valuable framework for explaining and forecasting health behaviors [15]. in contrast One of the theories utilized for anticipating behavior and executing educational interventions is the theory of planned behavior, which predicts an individual's intention to undertake a given behavior [22, 23]. According to this theory, three characteristics (attitude toward behavior, subjective norms, and perceived behavioral control [24]) can predict behavioral intention. This idea can explain nearly 40% of the correlations between intention and health behavior [25]. As a result, this model has the potential to construct instructional interventions for modifying unhealthy habits [26]. Because of the importance of preventive behaviors in osteoporosis prevention, TPB was used in this investigation. Given the rising prevalence of osteoporosis in Iran and the efficacy of preventive measures, it is critical to prevent this disease through educational interventions based on appropriate models. Developing and implementing a well-designed and comprehensive educational program is an important step in planning educational interventions and improving preventive behaviors. On the other hand, due to increased life expectancy and the incidence of osteoporosis, as well as a lack of understanding about osteoporosis in most women. The purpose of this study is to determine the effect of an educational intervention based on the theory of planned behavior on the behavioral responses of premenopausal women in the prevention of osteoporosis in Fasa, Iran.

## Methods

A complete list of helth centers of Fasa city, Iran was then prepared, of which two helth center were randomly selected using a random number table. The helth centers were subsequently assigned to the experimental and control groups by tossing a coin. Then, a total of 100 women aging from 35 to 55 years was randomly selected from each helth center.

Using the formula below 95% confidence level and 90% power and the rate of change after 2 weeks, a 22% reduction in the intervention group and 22% reduction in the control group in the study of Olson et al. [27], The minimum sample size in each group was calculated to be 92 people. Which was selected for the possibility of losing 100 participants in each group.

$$n = \frac{{\left(Z_{{1 -\alpha/ 2}} .\sqrt {2\overline{P}(1 - \overline{P})} + Z_{1 - \beta } .\sqrt {P_{1} (1 - P_{1} ) + P_{2} (1 - P_{2} )} \right)^{2} }}{{d^{2} }}$$

Also, the objectives of study were explained to them and. The first was obtaining clearance from the Research Ethics Committee. After determining the centers, sampling was done. The researcher referred to these centers and selected the required number of samples. The inclusion criteria were willingness to participate in the study, age 35–55 years, No physical or mental disabilities(they are not menopausal), ability to speak, perceive, and learn. The exclusion criteria were reluctance to continue the study and missing two educational sessions in a row. First, the purpose of the study was fully explained to the participants. After this phase, informed consent was obtained from the participants. Pre-test was conducted for both groups using a researcher-made questionnaire that were completed independently by the participants This questionnaire was developed according to recommendations [15, 27, 28] for “Constructing a Theory of Planned Behavior Questionnaire” [26] using valid and reliable literature.

The first section of questionnaire included demographic information such as age, BMI, educational level, marital status, job status, number of accouchements, lactation status, smoking, family history in osteoporosis and history in a specific disease. The second section included questions about the constructs of theory of planed behavior including questions about knowledge (6 questions, score range 6–48), attitude (16 questions, score range 16–128), subjective norms (20 questions, score range 20–160), perceived behavioral control(18 questions, score range 18–144), behavioral intention (9 questions, score range 9–72) (receiving calcium, receiving vitamin D and physical activity) in 8-point Likert scale from 1 to 8. from “completely disagree” with the score of 1 to “completely agree” with the score of 8.

An example of a Perceptions question in written form during baseline data collection was:

I know how much calcium I need every day to help prevent osteoporosis. Strongly Disagree Strongly Agree

I----I----I----I----I----I----I----I

The participant wrote an “X” between the corresponding marks on the line that best matched her response.

The third section of questionnaire included a check list (27 items) of amounts of nutrients eaten and Duration of physical activity about performance. Questions about nutrition performance evaluated the type and amount of nutrients eaten by subjects in previous week (scoring from 0 to 27). Exercise questions included 7 questions on the duration and type of walking (easy, moderate and heavy) in the week before the test based on the received guidelines (scored from 0 to 21). Participant’ performance was recorded through self-reporting answers.

To evaluate the validity of the questionnaire items, the impact index item higher than 0.15 and content validity index above 0.79 were considered.In order to determine the content validity, twelve specialists, and professionals (outside the research team) in the field of health education and health promotion (n = 10), orthopedic (n = 1), and biostatistics (n = 1) were consulted. Then, according to the Lawshe table, items with a content validity ratio (CVR) higher than 0.56 were considered acceptable and retained for the subsequent analysis [29].To assess the reliability of the instrument, a cross-sectional study was conducted on 40 females aged 35–55 years old referring to healthcare centers of Fasa city;iran. Then, the reliability of the instrument was assessed using the internal consistency method. The overall Cronbach’s alpha was 0.89 [By using Lawshe’s table index, items with CVR value higher than 0.56 were considered acceptable and retained for subsequent analysis.]. Moreover, the Cronbach’s alpha was [knowledge:0.87, attitude:0.86, subjective norms:0.80, and perceived behavioral control: 0.85, 0.82 for intention, 0.79 behavior].

Educational intervention for experimental group included 6 educational sessions for by giving as follow (Table 1): 2 weeks (Due to the effect of educational intervention, we also examined 2 weeks after the intervention to evaluate the effect of education based on the model, considering that the educational materials were not forgotten, Based on this, it is possible to conduct periodic trainings so that the content is routinely given to people and becomes part of their behavioral patter) and 2 months after educational intervention, experimental and control groups filled out the questionnaire. In order to evaluate bone density, experimental group was introduced to bone density evaluation center in Fasa city; iran and results were recorded.

**Table 1:** Description of educational sessions in experimental group

Session number	Content and strategy	Educational methods and materials/educator/learner assignment/time	Construct	Evaluation
First session	That was about osteoporosis, symptoms, complications and diagnosis	Presentations, group discussion, asking and answering questions and using educational posters, pamphlets, films and PowerPoints/researcher this study/ all paticipand of experimental group/55–60 min	Knowledge and atitude	Campare Pretese (before intervention) and posttest (2 weeks ans 2 month after intervention)
Second session	That was held with the presence of doctor, health center officials and one of family members as subjective norms. Also, a 55 years old woman suffering from osteoporosis was invited to talk about her disease, risk factor, symptoms and diagnosis		Subjective norms	
Third and fourth sessions	The role of nutrition in prevention from osteoporosis, benefits and barriers for following diet, proper nutrition programs, self-efficacy, perceived behavioral control for following appropriate diet based on presented pattern and recorded activities in determined forms were explained		Perceived behavioral control	
Fifth and sixth sessions	The role of proper exercising, walking, benefits and barriers for doing exercises, intention, type of exercises, perceived behavioral control and recorded results were explained		Doing exercises, intention, type of exercises, perceived behavioral control

**Table 2:** Demographic information of studied participants (n = 200)

Variable	Experimental group (n = 100)		Control group (n = 100)		P-value
	Number	Percentage	Number	Percentage	
*Job*					
Employed	18	18	15	15	0.235
Housewife	82	82	85	85	
*Educational level*					
Illiterate	4	4	2	2	0.197
Elementary	18	18	18	18	
Guidance School	33	33	30	30	
High school	36	36	35	35	
University	9	9	15	15	
*Marital status*					
Single	14	14	12	12	0.244
Married	77	77	75	75	
Divorced	4	4	6	6	
Widow	5	5	7	7	
*Lactation status*					
No	88	88	90	90	0.655
Yes	12	12	10	10	
*Smoking*					
No	98	98	99	99	0.678
Yes	2	2	1	1	
*Family history in osteoporosis*					
No	91	91	90	90	0.562
Yes	9	9	10	10	
*History in a specific disease (Like diabetes, cardiovascular disease,..)*					
No	85	85	89	89	0.712
Yes	15	15	11	11	

Variable	Mean $\pm \mathrm{SD}$	Mean $\pm \mathrm{SD}$	P < 0.05
Age	43.39 $\pm$ 5.20	42.94 ± 5.52	
BMI	23.15 $\pm$ 3.45	22.97 $\pm$ 3.71	
Number of accouchements	2.84 $\pm$ 1.68	2.77 $\pm$ 1.53	
Bone density T-Scores of femoral area and spinal cord	0.125 $\pm$ 1.232	− 0.238 $\pm$ 1.124	

*Chi-square was used for qualitative variables and t-test was used for quantitative normal variables to investigate homogeneity

Data were analyzed by SPSS-22 software. For evaluating quantitative variables, average score and standard deviation and for evaluating qualitative variables, frequency and frequency percentages were used. Also, Chi-square test, independent t-test, paired t-test and Repeated Measures ANOVAwere used.

## Results

Demographic variables are presented in Table 2.

Before educational intervention, there were no significant differences between experimental and control groups in knowledge, attitude, subjective norms, perceived behavioral control, behavioral intention, nutrition performance and physical activity. However, 2 weeks and 2 months after educational intervention, significant enhancement was observed in experimental group in each construct compared to control group (Table 3).

**Table 3:** Mean and standard deviation of TPB model structures in the intervention and control group before and after intervention (n = 200)

Variable	Group (N = 100)	Before intervention M $\pm$ SD	Two weeks after intervention M $\pm$ SD	Two months after intervention M $\pm$ SD	P-value
Knowledge	Experimental	18.29 $\pm$ 4.32	31.12 $\pm$ 4.20	39.04 $\pm$ 4.10	0.001*
	Control	19.14 $\pm$ 4.44	27.55 $\pm$ 4.18	36.65 $\pm$ 4.23	0.001*
	P-value	0.276	0.001**	0.001**	
Attitude	Experimental	54.17 $\pm$ 9.62	89.32 $\pm$ 9.22	98.57 $\pm$ 9.13	0.001*
	Control	52.87 $\pm$ 9.64	55.16 $\pm$ 9.08	57.32 $\pm$ 9.12	0.194
	P-value	0. 308	0.001**	0.001**	
Subjective norms	Experimental	40.28 $\pm$ 8.96	88.39 $\pm$ 8.84	122.57 $\pm$ 8.58	0.001*
	Control	43.08 $\pm$ 8.21	45.41 $\pm$ 8.73	48.56 $\pm$ 8.16	0.166
	P-value	0.247	0.001**	0.011**	
Perceived behavioral control	Experimental	47.24 $\pm$ 8.24	88.56 $\pm$ 8.38	120.15 $\pm$ 8.33	0.001*
	Control	46.31 $\pm$ 8.29	48.58 $\pm$ 8.39	51.30 $\pm$ 8.45	0.213
	P-value	0.177	0.001**	0.001**	
Behavioral intention	Experimental	32.22 $\pm$ 4.14	54.44 $\pm$ 4.72	60.26 $\pm$ 4.12	0.001*
	Control	30.96 $\pm$ 4.39	32.50 $\pm$ 4.22	34.58 $\pm$ 4.74	0.268
	P-value	0.362	0.001**	0.001**	
Nutrition performance	Experimental	12.09 $\pm$ 2.72	19.88 $\pm$ 2.56	24.14 $\pm$ 2.36	0.001*
	Control	11.67 $\pm$ 2.83	12.38 $\pm$ 2.61	14.29 $\pm$ 2.53	0.185
	P-value	0.428	0.001**	0.001**	
Physical activity	Experiential	9.12 $\pm$ 1.20	16.75 $\pm$ 1.42	18.94 $\pm$ 1.68	0.001*
	Control	9.93 $\pm$ 1.08	10.64 $\pm$ 1.46	11.42 $\pm$ 1.24	0.179
	P-value	0.433	0.001**	0.001**	

*Paired T-test, **independent t-test

## Discussion

The aim of this study was determining the factors affecting nutritional behavior and motor activity in the prevention of osteoporosis: Applying the theory of planned behavior in Fasa city, Iran. Training increased knowledge score in the experimental group. Other similar studies reported the increase of knowledge of participants about osteoporosis after educational intervention [8, 30–37]. Having knowledge about an especial issue as a background for creating or modifying attitude and taking appropriate actions is important [33].

Training increased attitudes score in the experimental group had enhancement. Discussing in groups and presenting positive and negative experiences caused participants to be more interested in patterning for taking prevention behaviors from osteoporosis. For promoting attitude, a picture book was given to participants that are in a good agreement with other studies about the effect of educational intervention on women’s attitude [38, 39]. The study of Gheisvandi et al. [40] reported significant enhancement in average score of attitudes of experimental group after intervention, indicating the effect of educational intervention based on theory of planned behavior on promoting the use milk and dairy for preventing osteoporosis.

After educational intervention, the average score of subjective norms had enhancement. The possible reasons of this enhancement in experimental group are the presence of doctor, health center officials and one of family members as effective people for performing prevention behaviors in educational sessions. Results of Gholamnia Shirvani et al. [41] Gheisvandi et al. [40] and Solhi et al. [39] indicated the enhancement of subjective norms of subjects after educational intervention.

The average score of perceived behavioral control of experimental group had enhancement. Solhi et al. [39], Gheisvandi et al. [40] and Parrot et al. [42] investigated the application of theory of planed behavior in educational intervention through sending Email about physical activity and Armitage et al. [25] studied the effect of educational intervention based on theory of planned behavior on physical activity of subjects. He showed the enhancement of perceived behavioral control of experimental group after educational intervention, compared to control group.

The average score of intention for preventing behaviors from osteoporosis had enhancement, which is in a good agreement with the results of Gheisvandi et al. [40] who investigated the effect of educational intervention based on theory of planned behavior on physical activity of participants and the study of Ramezankhani et al. [43].

The promotion of prevention behaviors from osteoporosis in nutrition performance is in a good agreement with the results of Vahedian Shahroodi et al. [32], Salimi et al. [44] and Gheisvandi et al. [40]. Theory-driven educational intervention, by emphasizing on effective factors on promoting prevention behaviors, such as having appropriate diet (using fruits, vegetables and foods containing calcium) in group discussion, presenting films, posters, pamphlets, explaining benefits, barriers and benefits of taking prevention behaviors and increasing behavioral control, caused the improvement of prevention behaviors from osteoporosis. Shabiri et al. [31] investigated the effect of consultation on prevention behaviors from osteoporosis in women who referred to health centers in Hamedan city, Iran. In his study, educational sessions held for experimental group in 4 weeks for 45–60 min based on gather consultation stages. His results indicated that, immediately and 2 months after educational intervention, the average score of experimental group in nutrition performance (using calcium) was significantly higher than control group. These results are in a good agreement with the results of Hsieh et al. [45] who reported the efficiency of educational programs on promoting prevention behaviors from osteoporosis in women. While in study of Shojaei et al. [33], the average score of using calcium in experimental and control groups was reduced after intervention. In his article “Educating prevention behaviors from osteoporosis: behavioral theories and receiving calcium”, Tussing et al. [46] indicated significant differences in received calcium by participants after intervention. By designing educational program based on health belief model and rational action theory, he could increase the amount of received calcium by subjects. In study of Shojaei et al. [33], before educational intervention, there were no significant differences in experimental and control groups in received calcium, however, after educational intervention based on health belief model in second stage (immediately after intervention), the average score of received calcium was enhanced in experimental group. Also, for continuing investigation, in third stage, (3 months after intervention), experimental and control groups were investigated again and the average of received calcium was reduced in both groups. However, there seemed significant differences in both groups in received calcium.

Educational intervention caused the promotion of prevention behaviors from osteoporosis in physical activity. In study of Niyazi et al. [34], educational intervention affected the studied issues and caused the improvement of physical activity of participants. In study of Tarshizi et al. [14], physical activity of experimental group was different from control group after educational intervention which was statistically significant. Mehrabbeik et al. [47] differences in physical activity after educational intervention which was in a good agreement. Results of studies [8, 38, 44, 48, 49]. In study of Vahedian Shahroodi et al. [32], there observed no significant enhancement in physical activity of experimental group which was probably due to the used questionnaire for evaluating physical activity of subjects. The International Physical Activity Questionnaire—Short Form (IPAQ-SF) was used which evaluated walking performance of participants. and medium and intense activities. Also, educational intervention had a great effect on promoting knowledge and constructs of theory of planned behavior and prevention behaviors from osteoporosis.

## Conclusion

Educational programs based on theory of planned behavior can cause the enhancement of knowledge and attitude of women about osteoporosis and promoting nutrition performance and physical activity for preventing this disease. Hence, health system managers should pay especial attention to important health issues such as osteoporosis. This theory can be used as a framework for designing and performing educational intervention for preventing osteoporosis and promoting women’s health.

One of the limitations was that, present results are related to women aging from 35 to 55 years who referred to health centers of Fasa city; iran, therefore, this study cannot be generalized to all women, especially elder women who are highly in exposure of osteoporosis. The other limitation was self-reporting answers of women about their prevention behaviors from osteoporosis.

## Data Availability

The datasets generated and/or analyzed during the current study are publicly available from the corresponding author on request.

## Abbreviations

DEXA: Dual energy X-ray absorptiometry
TPB: Theory of planned behavior
